# Supplementary material for: Rapid health impact assessment of COVID-19 on families with children with disabilities living in low-income communities in Lusaka, Zambia
Source: PLoS One. 2021 Dec 15;16(12):e0260486. doi: 10.1371/journal.pone.0260486 (PMC8673599; doi:10.1371/journal.pone.0260486)
Supplement: Attachment — Submitted filename: Review Comments to the Author PlosOne May21.docx [file pone.0260486.s002.docx]

Review Comments to the Author

This is a very important topic and it is great that the authors thought about doing this research in such a timely manner. Unfortunately however, I believe the paper needs significant revisions before it can be considered for publication.

My main concerns are with the methodology, data collection and the results and the lack of information provided in each of these sections that make me question the validity of the data.

Below I am outlining a detailed set of feedback for each section. Please note that his feedback is intended to support the development of the paper and preparation for future submissions.

**Introduction:**

Lines 49-51: The sentence starting with “Those with feeding..” seems a little out of place and context here as presented. Perhaps it can be presented as an example of a difficulty of challenging situation and include how this is more complicated by the pandemic and presence of Covid-19.

Line 54: It is unclear which type of “global Leaders” the authors are referring to. Politicians? Leaders in public health?

Lines 56-59: What do the authors hope the results to be used for? Specifying this more clearly would make it more effective. For example in terms of an post pandemic response? Or for future pandemics? For use during this current pandemic management? All of the above?

**Materials and Methods:**

Participants

Line 67: references provided in two formats- needs to be fixed.

Line 70-71: Did all the families who were approached accept to participate in the study? Did any one decline participation and did the authors have to select more names?

Line 72-73: Similar to above comment, did all CCGs accept request for participation or was there any who declined?

Line 74:Who were the health professionals? What kind of clinicians/practitioners were they? And again did all who were initially approached agree to respond?

Line 77: For additional details regarding the Kusamala+ should provide a proper title of a report or link as apposed to just a reference number.

Measures

Line 80:Trained “personal”- spelling mistake but also, trained in what? And how were they identified? How is the Catholic Medical Mission Board connected to the authors or get involved- are they were the authors are from?

Line 81: How were participants contacted and consented? How were the paper based surveys developed and by who?

Line 83-84: Whose demographic information was asked about from the families: the respondent? the child with the disability? both?

Line 89: the CCG were asked questions in the same areas as who? The families?

Line 91: Type should be “CCGs” not “CCGS”.Also, why were the CCGs and health professionals asked for their recommendations and not the families and caregivers who actually experienced the difficulties? Or did they provide recommendations without being asked?

Line 92-93: Why were the government officials who are the furthest removed form the lived experiences of the families of children with disabilities asked to reflect on the services and the gaps and needs as opposed to the families, the CCGs or the health professionals that work directly with the families.

**Analysis:**

It would have been good to know which type of thematic analysis process was used to analyse the qualitative data.

**Results:**

I would highly recommend including the types of disabilities that were experienced by the children.

Line 107: Given the population is children, even if it seems obvious should always include the scale/units for all categories as years so not confused with months for younger kids/infants.

Line 109: When discussing family members with symptoms, it would have been useful to know it they attempted to get tested, would have liked to be tested and were not able to due to various community or family related barriers?

Line 110: Who/what are examples of trusted sources? Clarification needed.
