# Supplementary material for: Rapid health impact assessment of COVID-19 on families with children with disabilities living in low-income communities in Lusaka, Zambia
Source: PLoS One. 2021 Dec 15;16(12):e0260486. doi: 10.1371/journal.pone.0260486 (PMC8673599; doi:10.1371/journal.pone.0260486)
Supplement: Attachment — Submitted filename: Response to Reviewers R1.docx [file pone.0260486.s003.docx]

Response to Reviewers

Please note that all edits in the paper were done in red font.

| Reviewer Comment | Response |
| --- | --- |
| Reviewer #1: Review Comments to the Author This is a very important topic, and it is great that the authors thought about doing this research in such a timely manner and they have made some decent justifications of why this is important in the discussion. Unfortunately, however, I believe the paper needs significant revisions before it can be considered for publication. My main concerns are with the methodology, data collection and the results and the lack of information provided in each of these sections that make me question the validity and usefulness of the data. Below I am outlining a detailed set of feedback for each section. Please note that his feedback is intended to support the development of the paper and preparation for future submissions. | Thank you for your support and review. We framed it as a short commentary, thus restricting too many details. I appreciate your feedback and look forward to your response to the revised version. |
| Introduction: Lines 49-51: The sentence starting with “Those with feeding..” seems a little out of place and context here as presented. Perhaps it can be presented as an example of a difficulty of challenging situation and include how this is more complicated by the pandemic and presence of Covid-19. | We agree and have edited the section with examples. |
| Line 54: It is unclear which type of “global Leaders” the authors are referring to. Politicians? Leaders in public health? | The language was changed to global health leaders and citations were added to the United Nations and the International Disability Alliance |
| Lines 56-59: What do the authors hope the results to be used for? Specifying this more clearly would make it more effective. For example, in terms of an post pandemic response? Or for future pandemics? For use during this current pandemic management? All of the above? | Helpful feedback. We modified the language to be more specific.  As governments and civil society continue to adapt their policies and programs in response to the pandemic, new strains, and vaccine distribution, it is critical to use information from the lived experience of children with disabilities and their families to combat existing inequities and inform policy solutions for this population of children. The pandemic is ongoing and mutating; therefore, the voices from the community can inform current COVID-19 pandemic policy and pandemics that will come in the future.  In the conclusion: While ambitious goals, these children are often hidden in society. This data provides an opportunity to illuminate the need to move children with disabilities to the forefront of decision-makers and the community of practice’s agenda. |
| Materials and Methods: Participants Line 67: references provided in two formats- needs to be fixed. | Thank you for catching that – EndNote mishap. |
| Line 70-71: Did all the families who were approached accept to participate in the study? Did any one decline participation and did the authors have to select more names? | This information was added to the manuscript. |
| Line 72-73: Similar to above comment, did all CCGs accept request for participation or was there any who declined? | This information was added to the manuscript. |
| Line 74: Who were the health professionals? What kind of clinicians/practitioners were they? And again, did all who were initially approached agree to respond? | This information was added to the manuscript. |
| Line 77: For additional details regarding the Kusamala+ should provide a proper title of a report or link as apposed to just a reference number. | This was corrected |
| Measures Line 80: Trained “personal”- spelling mistake but also, trained in what? And how were they identified? | The misspelling was removed and the sentence modified. “In October and November 2020, Catholic Medical Mission Board (CMMB) - Zambia staff with experience in data collection with families who have a child with a disability contacted…” We also noted that we trained the interviewers. |
| How is the Catholic Medical Mission Board connected to the authors or get involved- are they were the authors are from? | The introduction to the participants was altered providing more detail about CMMB and the relationship with St. Catherine University |
| Line 81: How were participants contacted and consented? How were the paper-based surveys developed and by who? | Kusmala+ has a roster of participants with phone numbers. The randomly selected families were contacted by phone and consented by CMMB staff. |
| Line 83-84: Whose demographic information was asked about from the families: the respondent? the child with the disability? both? | This was clarified in the paper. |
| Line 89: the CCG were asked questions in the same areas as who? The families? | This was clarified. |
| Line 91: Type should be “CCGs” not “CCGS”. Also, why were the CCGs and health professionals asked for their recommendations and not the families and caregivers who actually experienced the difficulties? Or did they provide recommendations without being asked? | This was corrected and addressed. |
| Line 92-93: Why were the government officials who are the furthest removed form the lived experiences of the families of children with disabilities asked to reflect on the services and the gaps and needs as opposed to the families, the CCGs or the health professionals that work directly with the families. | They were all asked about the services and gaps, each from their own point of view based on their position. The families were asked about their experiences and, as part of that, reported on their challenegs and needs; CCGs and health professionals reported on their observations of the community in which they live, and the government provided their opinions on what they think the gaps are. So, we were tying to assess perceived gaps at various levels. |
| Analysis: It would have been good to know which type of thematic analysis process was used to analyze the qualitative data. | We used Braun and Clark’s steps in thematic analysis. More details were added. |
| Results: I would highly recommend including the types of disabilities that were experienced by the children. | This was added |
| Line 107: Given the population is children, even if it seems obvious should always include the scale/units for all categories as years so not confused with months for younger kids/infants. | Good idea – that was adjusted. |
| Line 109: When discussing family members with symptoms, it would have been useful to know it they attempted to get tested, would have liked to be tested and were not able to due to various community or family related barriers? | We agree. Unfortunately, we did not ask this. |
| Line 110: Who/what are examples of trusted sources? | Another good question – it was the health facility and that was added to the paper |
| Clarification needed. Line 116: Figure one is meant to present the perceived negative impact of Covid-19, yet it includes improvements as well-perhaps title of table needs to be adjusted. | True – the word ‘negative’ was removed from the title. |
| Line 117-118: greater discussion or examples of the results would have been beneficial as to how families were greatly impacted in the various areas identified. | Several quotes were added for illustration |
| While examples of quotes are provided with some of the qualitative data- this is still limited and not substantial enough. Line 122: Where referring to school closures- perhaps need to make a distinction on how this has an impact that is different from non-disabled children for the children with disabilities who actually attended school. | Additional quotes have been added.  We did not collect data on non-disabled children for this rapid assessment so we cannot fully respond. |
| Line 124: Typo”3.2”. Also, it would have been good to provide a detailed break down of the numbers of the healthcare professionals and government officials and CCGs here as well for consistency with above section with families. In addition, providing demographics on this group would have been useful such as their roles/ posts, years of experience? Gender? | Additional data was added in the methods about who the health professionals and government officials are and their titles. |
| Line 126: Table 1- While the themes seem to be derived from all participant groups. All examples of quotes are from family members- more examples and from other participants would be of great value. Also including or acknowledging that in some cases the result may have not been what was expected. | This table focuses on the families’ perceptions. Additional detail was added to the results to illustrate examples and quotations across those interviewed. |
| Discussion Line 130: While we know the pandemic will have long lasting effects- we have not yet experienced them and so the sentence may need to be slightly adjusted and the impact of course is on all, including families and children with disabilities. | You are correct – we adjusted the language |
| Line 154: The sentence about stigma is very general and needs to be tied to the impact of the Pandemic given the focus of the paper. | The discussion was updated - appreciate the feedback |
| Reviewer #2: This is a well written and conducted study and interesting paper dealing with a hot topic. Indeed, little is known on how COVID-19 pandemic might have affected families with children with disabilities from low to middle income countries. The present study sheds light on the perceived impacts of COVID-19 for children with disabilities and their families from the parents, community caregivers, health professionals, and government officials. The rationale, relevance, and methodology/descriptive analyses of the study are adequately addressed. Main limitations include the small sample size and the population included in the study were only from the Kusamala+ program. I consider some adjustments to the manuscript to be required: | Thank you |
| 1. The abstract would be designed in a structured way. Some key findings are missing. | The abstract was adjusted and additional results added |
| 2. To better frame the context of the study and the results, it would be informative for the reader to specify when the COVID-19 outbreak reached Zambia –especially the location of the study (Lusaka)– and if there were any restrictive measures or localised lockdown taken by the government or the education system that affected the population of the study. It is suggested to briefly introduce the context in a separate section. That is, first Zambia and then Lusaka should be introduced to the readers. It is also better for the authors to mention the time range of quarantine/confinements in Zambia. Also, has Lusaka province been subject to national or local restrictions? | Additional information was added to provide the readers more context. |
| 3. Page 7, lines 135-137: The paper states that "...to our knowledge, no work has been published on the impacts of COVID-19 on children with disabilities living in low-income communities in Zambia." The authors point out that no research has been done/published on the impacts of the COVID-19 on children with disabilities in Zambia. This sentence may not be completely correct and by searching it can be found that some studies have been done in Zambia about the impact that COVID-19 pandemic had on children with disabilities and their families. It is also better to refer to the surveys conducted in Zambia and to present its important findings and shortcomings. DOI: <https://doi.org/10.1186/s40359-021-00583-w> <https://doi.org/10.12688/hrbopenres.13077.2> | Thank you so much for this information. We have incorporated the information and changed our language. The article by Linehan was for a planned interview so there were no results. |
| 4. Please report the types of disability in participants, especially given the emphasis on disabled children population. | This was added |
| 5. Interview guide: Can you please explain the domains of questions asked, the purpose of the questions, and give some example questions? | Additional detail was provided over who interviewed and how the questions were designed per another reviewer. We no longer refer to the interview guide. It was a list of the questions and some prompts. I am not sure it is needed at this point, but am open to suggestions |
| 6. About the discussion, I suggest that you could make a more in-depth discussion regarding the role of community caregivers, health professionals, and government officials. | Good idea. This was added. |
| 7. It's suggested to follow a similar manner for spelling: toward/towards, among/amongst etc. | Corrected, thank you |
| Reviewer #3: This manuscript highlights important issues families with children with disabilities in sub-Saharan Africa are struggling with due to the COVID-19 pandemic.  The data presented is very interesting, however the manuscript lacks academic rigor. | Thank you – your comments helped make this paper stronger |
| The introduction and discussion mostly consists of references to UN reports and a few academic papers. There is a need for a thorough literature review of papers already published on the impact of COVID-19 on persons with disabilities in low income countries (the Disability Inclusive Response paper by Banks et al could be a helpful starting point), and more specifically children with disabilities in African countries. Battle, Coker, Kamga, Mbazzi, Mcinney, Mupaku, Ned, Samboma to mention a few have earlier highlighted the impact COVID has had on adults and children with disabilities in sub Saharan Africa. | We were able to find many of these references and a few others. Thank you for the recommendation and the introduction was expanded and more inclusive |
| In the method section I would like to see more information on the survey design, e.g. who designed the survey questions, were families of children with disabilities involved in this process? What was the background of the interviewers, it is mentioned they were trained personnel, did they have any research training, were they Zambian, what language did they use to conduct the interviews. How were phone interviews recorded and who transcribed and where needed translated the recordings? There is mention of the use of Google forms, how were GDPR guidelines respected and was data kept safe? The data analysis followed some of Braun’s steps, though a full analysis plan and reference is missing. Were the findings checked with participants? What kind of referral system / care was in place for participants who shared serious health or food security concerns? It may be helpful to consult the COREQ and SRQR guidelines for qualitative study reports when revising their paper. | -The methods section was expanded to include more information  -the interviews were done in the local language of Nyanja. The data collectors were bilingual and completed the interviews with forms that included check boxed (English) and transcribed any responses from Nyanja to English upon transcription. Interviews were not recorded as they followed a survey approach  -Google forms were available only to data collectors, researchers and the Monitoring and Evaluation Coordinator at CMMB Lusaka. In addition, data was confidential for researchers because ID numbers were used limiting identifiability.  -Additional detail was added to the analysis section  -Using the guides you suggested, we have improved the reporting |
| The text in the findings section is limited to one page. The table and figure show interesting quotes and themes. It would be interesting to describe these in more detail in the text and provide other quotes to support the themes too. The title indicates the participants are from 3 low income communities but data is not segregated by these communities. Could the authors clarify the rational of including this specifically in the title and/or explain more about the differences between the 3 communities if any in the findings section?  Similar to the introduction, the discussion section sets out important points, but is lacking references to policies and statements made around COVID-19 and disability in low income countries, and particularly sub-Saharan Africa over the past year. | Additional results were added in the results section. As the data was aggregated, the names of the communities are less important for the audience. The sample was much too small to present by community.  Other reviewers agreed with you and more was added here, too. |
| I hope the authors will consider revising the manuscript, as the data give interesting insights and recommendations from a continent that is greatly affected by the pandemic, but remains underrepresented in the academic literature as well as media reports. | Thank you – couldn’t agree more. |
